# High prevalence of ghost rates in transparency in coverage data

**DOI:** 10.1093/haschl/qxaf212

**Published:** 2025-11-12

**Authors:** David B Muhlestein

**Affiliations:** Simple Healthcare

## Introduction

In 2019, an executive order^1^ required hospitals and insurers to release negotiated rates for services—hospitals in 2021^2^ and insurers in 2022.^3^ In 2025, an additional executive order confirmed this requirement and expanded efforts to make drug prices available.^4^ These transparency efforts are intended to increase competition, innovation, and value in healthcare by making prices public.

A major challenge with the transparency in coverage (TiC) data, which covers all inpatient, outpatient, postacute, and physician offices, is that many rates relate to providers and groups that do not provide those services. For example, a psychiatrist will never perform a heart transplant, but insurers may report a negotiated rate for that service. These negotiated rates—known as ghost rates—undermine the goals of transparency data.^5,6^

Ghost rates can significantly increase the size of the data and make evaluation more difficult as researchers and analysts do not know if they should include each reported rate in their analysis, resulting in distrust of the TiC data. This research letter provides estimates of how common ghost rates appear in TiC data from 61 insurers including 3 national commercial insurers (CVS/Aetna, Cigna, and UnitedHealthcare), and 58 Blue Cross Blue Shield insurers (including plans sponsored by Elevance/Anthem and Health Care Service Corporation).

## Methods

I identify ghost rates at the group level, the individual provider level, and across networks and calculate the percentage of reported negotiated rates that are ghost rates. The process to identify ghost rates is composed of 3 steps. (1) Identify the types of providers that perform various billing codes utilizing claims data from Medicare and multiple state All Payer Claims Databases. (2) For each individual provider—based on their National Provider Identifier (NPI)—determine if they are likely to perform a service based on either having performed that claim previously (from claims data) or if their specialty is likely to perform that service. (3) Since negotiated rates are provided at the provider-group level, determine whether any provider within that group is likely to perform each billing code; if no providers in the group are likely to perform the code that negotiated rates is a ghost rate. A subanalysis evaluates the prevalence of ghost rates for only the 100 most common billing codes; while there are more than 12,000 billing codes in the data, these 100 represent more than half (56.5%) of claims. A more complete description of the methodology is available in the Supplementary Appendix.

## Results

**Figure 1.**
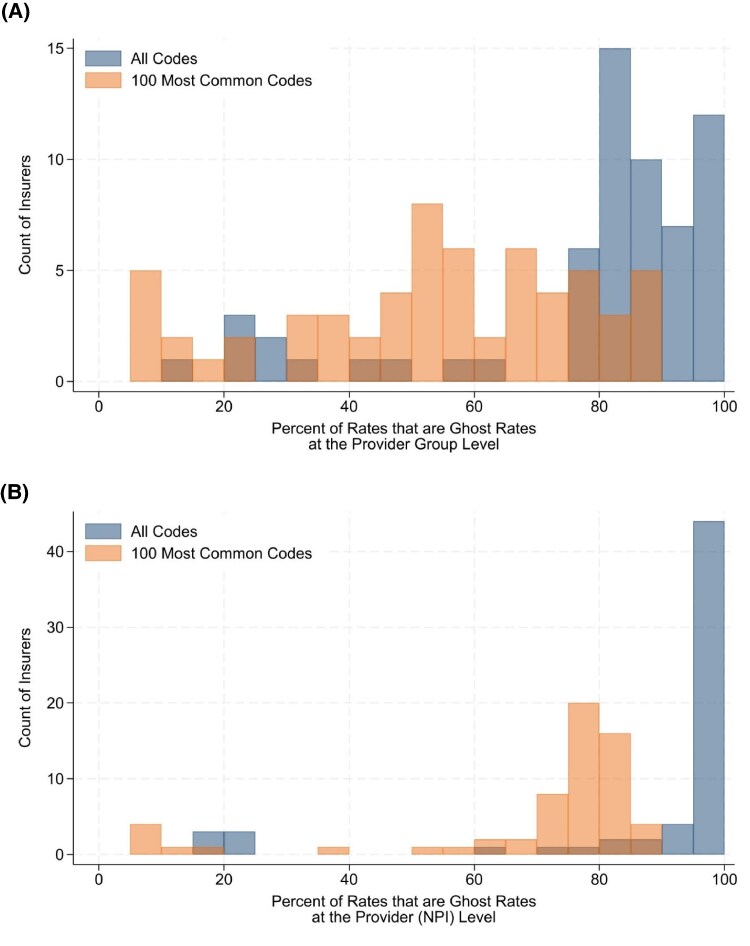
Percent of negotiated rates that are ghost rates. (A) Percent of rates that are ghost rates at the provider-group level. (B) Percent of provider-to-billing code pairs that are ghost rates.

Figure 1A contains a histogram of the percent of rates for each insurer that are ghost rates and the percent of the 100 most common billing codes that are ghost rates. The median insurer's data consisted of 84.3% ghost rates with the mode (15 insurers) having from 80% to 85% ghost rates; the range was 12.6% to 97.3%. Across a combination of all 61 insurers, 91.8% of all the negotiated rates were ghost rates (3 153 469 476 out of 3 433 560 471). The 100 most common billing codes represent 1.1% of the data; across the 61 insurers, 70.3% of the negotiated rates were ghost rates with a range of 6.3% to 89.8%.

All TiC negotiated rate data are presented at the provider-group level, but includes individual NPIs, allowing me to calculate how many of the included NPIs have ghost rates. Figure 1B provides a histogram of the percent of all providers (based on NPIs) and their corresponding billing rates that are ghost rates. Across all 61 insurers, the median insurer had 95.7% ghost rates, with a range of 16.8% to 98.6%. Combining all 61 insurers, 95.4% of provider-to-billing code pairs were ghost rates (112 297 416 974 out of 117 682 739 557 pairs). The 100 most common billing codes represent 1.1% of the provider-level data; across the 61 insurers, 75.5% of the negotiated rates were ghost rates with a range of 5.3% to 89.2%.

## Discussion

The goal of the TiC data is to empower the healthcare industry to make better informed decisions. However, with over 90% of all listed rates across these 61 insurers being ghost rates, it is far from ideal to achieve that purpose. For the TiC data to become actionable, it needs significant processing and cleaning, or working with a third party that can provide clean data.

The prevalence of ghost rates is lower for the most common billing codes, as more providers are likely to perform those codes. For example, most specialties of providers regularly perform office visits, so very few negotiated rates for office visits are ghost rates. However, the 100 most common billing rates, which represent over half of all claims, only account for 1.1% of the negotiated rates, and over 70% were still ghost rates.

There are 2 competing goals with the data that are reflected with ghost rates. First, the goal of providing as much information as possible; in this case, every negotiated rate that the payer has contracted with every provider. Second, providing data that is accurate and easy to act upon; in this case, data that is for providers who currently perform, or may perform, the service. The way to address both issues is to require insurers to include volume information at the network-level that indicates how many times each provider group has been paid for that code over the previous year. With that data, it would be straight-forward to remove low-volume or no-volume providers, allowing accurate lists of providers rendering those services. Without volume data, there is not a simple approach to remove ghost rates and most analysts will be reliant on data vendors to identify ghost rates. Given the size and structure of the data, it is unlikely that the TiC files will ever readily be accessible to patients and unsophisticated employers, so the design should be focused on optimizing the data for analysts and researchers.

Ghost rates are very common and represent the majority of TiC data, limiting its usefulness without significant cleaning and processing. To maximize the potential of the TiC data, CMS should require payers to include volume information, allowing users to quickly identify ghost rates.

## Supplementary Material

qxaf212_Supplementary_Data
