# Supplementary material for: High prevalence of ghost rates in transparency in coverage data
Source: Health Aff Sch. 2025 Nov 12;3(11):qxaf212. doi: 10.1093/haschl/qxaf212 (PMC12631121; doi:10.1093/haschl/qxaf212)
Supplement: qxaf212_Supplementary_Data [file qxaf212_supplementary_data.zip › Appendix.pdf]

Technical Appendix

Methodology

Appendix Figure 1 provides a flow chart of the process to identify ghost rates. This involves identifying ghost rates at the individual level, applying that output to groups, and then calculating total prevalence of ghost rates.

Appendix Figure 1: Flow Chart of Ghost Rate Identification Process

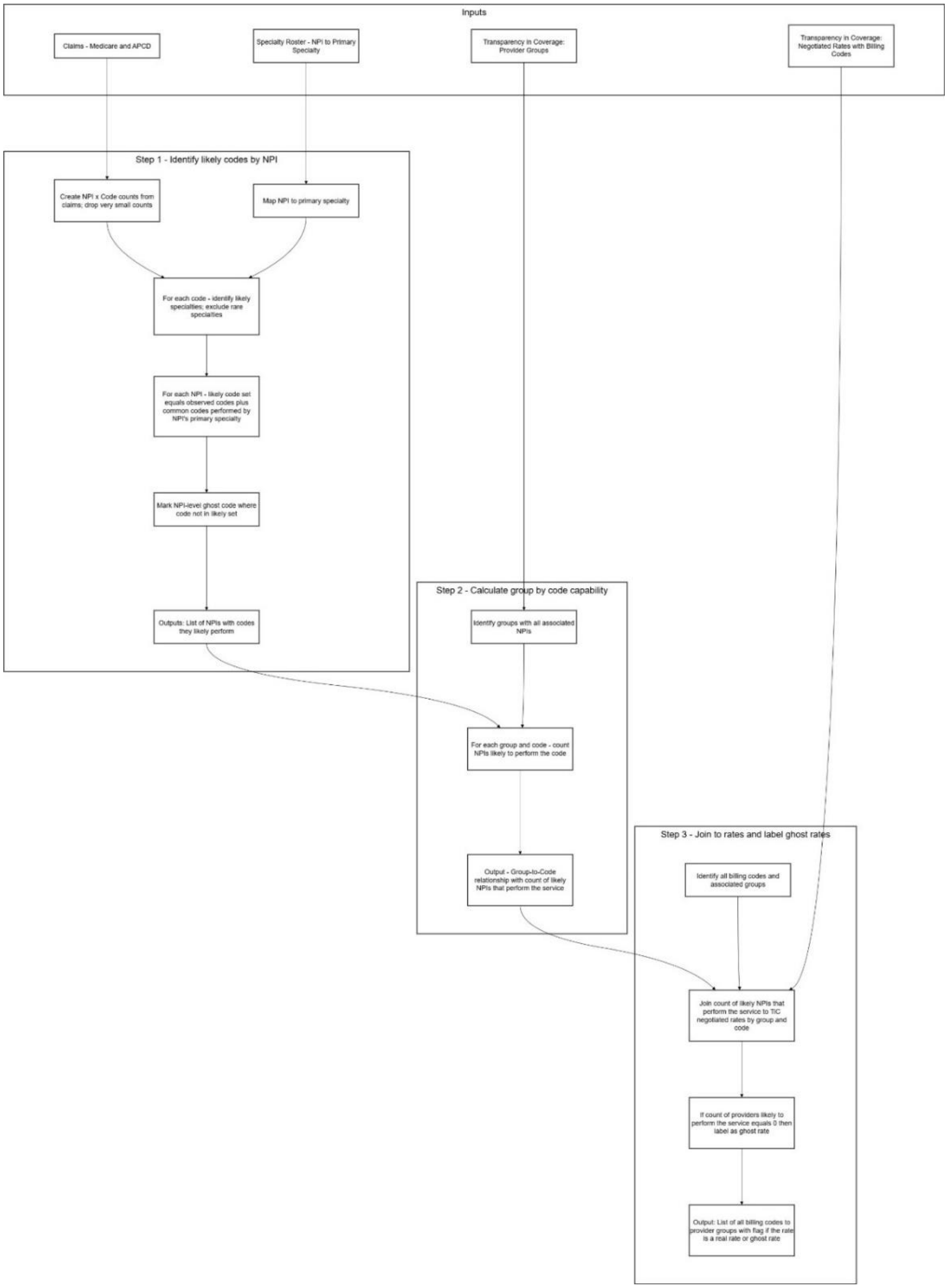

Individual Provider Ghost Rates. As a ghost rate is a code that a provider is unlikely to perform, this calculation must begin at the individual provider-level. Within the TiC data, individual providers are identified by National Provider Identifier (NPI) numbers, so ghost rates are identified at the NPI-level. Identification of ghost rates is based on claims data – both from Medicare claims and All-Payer Claims Databases (APCD) data. Ghost rates are evaluated at the billing code-level; I use Healthcare Common Procedure Coding System (HCPCS)[1] and Medicare Severity Diagnosis Related Groups (MS-DRG)[2] codes.

Determining which codes are ghost rates begins with identifying providers that have previously performed these services which results in a series of NPI-to-billing code relationships. Claims data can be unreliable, so I exclude NPI-billing code relationships with very small counts of claims which may be mistakes. I then match the NPI to its primary specialty and identify the specialties most likely to do each code. Similar to claims data, specialty data is not always accurate, so I exclude the specialties that very rarely bill for the code. The output is a list of which specialties are likely to perform each billing code.

Next, I identify, at the NPI-level, which billing codes the provider is likely to perform. I first include all the billing codes that they have performed based on claims data (excluding very low-volume billing codes that may be a mistake). I then add in the billing codes that their primary specialty tends to perform. The output is a list of every NPI and each of the billing codes that they currently perform or, based on their primary specialty, have the likelihood to perform in the future. Any code that is not on this list that contains a rate in the TiC data is a ghost rate for that provider. See Appendix Figure 1, Step 1. Importantly, this approach errs on being more inclusive of providers who may perform the service in the future based on their specialty, and not limiting to providers who have previously performed the service.

Provider Group Ghost Rates. Ghost rates are reported negotiated rates where nobody in the provider group is likely to perform the service, so this analysis is done at the provider group-level. Within the TiC data, provider groups are defined as combinations of one or more tax identification numbers (TINs), each with one or more NPIs. Different provider groups may include the same TINs which can be defined multiple times in slightly different ways throughout the file; for example, there may be a TIN that covers six providers, four of which are medical doctors (MDs), and two of which are nurse practitioners (NPs); the insurer may then define provider group 1 as the TIN with the four MDs, and provider group 2 as the same TIN with the two NPs, because they may have different negotiated rates. This organization is important as in this example a specific rate may be a ghost rate for provider group 1 but not group 2, or vice versa.

I first identify every NPI within each provider group and merge in all the billing codes that each NPI has performed or is likely to perform in the future. I then sum the count of providers, at the group level,

that are likely to perform each service. The output is a provider group-to-billing code relationship. See Figure 3, Step 2.

I then merge each provider group-level negotiated rate from the TiC data to identify the count of providers likely to perform each billing code that has a negotiated rate. If there are no providers in the group that are likely to perform the service, then that negotiated rate-to-provider group relationship is a ghost rate. See Figure 3, Step 3.

Prevalence of Ghost Rates. Calculating the prevalence of ghost rates is done at the network-level. Insurers may have multiple networks, but for this analysis I limit the analysis to the most common network (generally a preferred provider organization plan) for each insurer. I downloaded the original files for each insurer directly from their website in Quarter 3 of 2025. Some of the insurers release their network rates files in sub-files, in which case I included each of the subfiles and combined them into a single analysis file.

I started with every negotiated rate-to-provider group combination in the data and then remove certain subsets of the data. First, I remove custom billing codes (i.e., payer-specific codes that are not HCPCS or MS-DRG codes) which do not have a basis on which to determine which providers perform the service. I then limit the type of negotiation arrangement to rates that are based on a negotiated rate, derived rate or fee schedule[3] with a dollar value. I can then calculate, for each payer, the percent of total rates that are ghost rates.

Each provider group includes NPIs that are part of the group. By extending the data to the NPI level, I can calculate the percent of individual provider rates that are ghost rates.

Most common billing codes. I determined the most common billing codes by referencing the same Medicare and APCD claims data and calculated how often each billing code was performed/billed. I created a rank-ordered list, and included the 100 most-common billing codes to assess the same prevalence of ghost rates for these more common codes.

References

1. Centers for Medicare & Medicaid Services. Healthcare Common Procedure Coding System (HCPCS). Accessed August 13, 2025. https://www.cms.gov/medicare/coding-billing/healthcare-common-procedure-system

2. Centers for Medicare & Medicaid Services. MS-DRG Classifications and Software. Accessed August 13, 2025. https://www.cms.gov/medicare/payment/prospective-payment-systems/acute-inpatient-pps/ms-drg-classifications-and-software

3. CMS Price Transparency Schema. GitHub. Accessed August 19, 2025. https://github.com/CMSgov/price-transparency-guide/tree/master/schemas
